# Precipitation Polymerization-Based Molecularly Imprinted Polymers: A Novel Approach for Transdermal Curcumin Delivery

**DOI:** 10.3390/polym16243456

**Published:** 2024-12-10

**Authors:** Muhammad Cholid Djunaidi, Viona Resda Putri, Nesti Dwi Maharani, Retno Ariadi Lusiana, Parsaoran Siahaan, Sunarno Sunarno

**Affiliations:** 1 Department of Chemistry, Faculty of Science and Mathematics, Diponegoro University, Jl. Prof. Soedharto SH, Tembalang, Semarang 50275, Indonesia; vionaresda17@gmail.com (V.R.P.); nesti.dwimaharani@gmail.com (N.D.M.); retno.lusiana@live.undip.ac.id (R.A.L.); siahaan.parsaoran@live.undip.ac.id (P.S.); 2 Department of Biology, Faculty of Science and Mathematics, Diponegoro University, Jl. Prof. Soedharto SH, Tembalang, Semarang 50275, Indonesia; sunzen07@gmail.com

**Keywords:** curcumin, molecularly imprinted polymer, transdermal drug delivery

## Abstract

This research describes the synthesis and characterization of a molecularly imprinted polymer (MIP) as a candidate for the transdermal delivery of curcumin. The MIP was synthesized through precipitation polymerization using methacrylic acid as the functional monomer and ethylene glycol dimethacrylate as the cross-linking agent. MIP characterization studies were conducted using SEM-EDX and FTIR spectroscopy to determine the morphology and interaction between curcumin and polymers. The MIP obtained through precipitation polymerization was in the form of a fine powder with a surface morphology resembling a collection of small granules with a uniform shape. The adsorption capacity of the MIP follows the Langmuir adsorption isotherm model, with a maximum capacity of 4.239 mg/g, which is greater than that of the NIP (3.219 mg/g), resulting in an imprinting efficiency of 1.317. The percentage of curcumin released from the MIP after 8 h was 41.26%, which is lower than that from the NIP, at 51.50%. The drug release kinetics study follows the Higuchi model, indicating drug diffusion from the polymer matrix. Imprinting on the MIP can modify drug diffusion from the polymer matrix, resulting in a reduced release rate in the MIP. Therefore, the MIP can be considered a candidate for the controlled transdermal delivery of curcumin.

## 1. Introduction

Curcumin, or diferuloylmethane, is one of the active biological components of the turmeric rhizome plant. It has various benefits in the health sector because of its pharmacological activity. However, curcumin has the weakness of low solubility in water, which results in incomplete absorption in the digestive tract [1]. In addition, the stability of curcumin is quite poor because it is easily degraded at physiological pH if not protected by a suitable carrier [2]. Therefore, appropriate candidate carriers and delivery methods are needed to achieve optimal therapeutic results.

Due to the limitations of the oral delivery of curcumin, the transdermal route can be used as an alternative delivery system. Transdermal drug delivery systems refer to the systematic application of drugs to the surface of the skin to allow the transfer of the drug into the blood vessels. The transdermal route offers advantages compared to oral or intravenous methods, including easy application, comfortable use for sensitive patients such as children and the elderly [3], preventing drug exposure in the gastrointestinal tract where the drug may degrade, avoiding the first-pass effect where the drug molecule undergoes metabolism at specific sites (leading to a reduced drug concentration after reaching the target), and having the ability to control drug release activity [4].

Molecularly imprinted polymers (MIPs) are synthetic materials prepared through molecular imprinting techniques, with the advantage of selectivity and specific affinity for the template molecule so that MIP can be used as a carrier in drug delivery systems [5]. MIPs have good physical and chemical stability under extreme conditions such as extreme pH, high temperature, high pressure, and the presence of organic solvents [6]. In addition, MIPs also have the advantages of increased drug loading, easy preparation, and cost effectiveness [7]. Previous research has shown that MIPs can be applied as a polymer matrix in transdermal delivery, where they are able to increase stability and control drug release [8].

MIP synthesis can be achieved through several methods, including the precipitation method [9]. The precipitation method is a simple polymerization method that involves excessive amounts of porogen so that the resulting mixture is more homogeneous and the resulting polymer has a controlled morphology [10]. Previous research succeeded in synthesizing MIP nanoparticles for the controlled release of trinitroglycerin using the precipitation polymerization method, which showed that the obtained polymer matrix had a uniform shape and size and could be applied for controlled drug delivery in biological fluids due to selective binding [11].

Based on this background, in this research a molecularly imprinted polymer was synthesized for the controlled delivery of curcumin via transdermal delivery. This research includes the synthesis and characterization of curcumin-imprinted MIPs with methacrylic acid (MAA) as the functional monomer, ethylene glycol dimethacrylate (EGDMA) as the cross-linking agent, benzoyl peroxide (BPO) as the initiator, and acetonitrile as the porogen via precipitation polymerization. The use of MAA and EGDMA in MIP synthesis was due to their biocompatible and non-toxic nature [12]. The obtained MIP samples were tested for their adsorption capabilities and characterized using Scanning Electron Microscope-Energy Dispersive X-ray (SEM-EDX) analysis and Fourier Transform Infrared (FTIR) spectroscopy. In addition, to determine the percentage of curcumin released from the sample, an in vitro test was carried out in a phosphate buffer solution (PBS pH 7.4).

## 2. Materials and Methods

### 2.1. Materials

The following materials were purchased: curcumin, methacrylic acid, ethylene glycol dimethacrylate, and benzoyl peroxide were purchased from Sigma Aldrich, Darmstadt, Germany; acetonitrile p.a., methanol p.a., glacial acetic acid p.a., ethanol p.a., Na_2_HPO_4_ pa, NaH_2_PO_4_ p.a., propylene glycol, and 0.45 µm Millipore membrane filter were purchased from Merck, Jakarta, Indonesia; nitrogen and demineralized water were purchased from Bratako Chemika, Semarang, Indonesia; 90 mm filter paper and pH paper were purchased, Macherey-Nagel, Düren, Germany; and wrap and aluminum foil were purchased from Klin Pak, Bogor, Indonesia.

### 2.2. Tools and Instruments

The following tools and instruments were used in the experiments: a set of laboratory glassware (Herma and Pyrex), spatula, Ohaus CP 214 analytical balance, hotplate stirrer (IKA C-MAG HS7, Staufen, Germany), magnetic bars, shaker (DLAB SK-O180-S, Senai, Malaysia), oil bath, syringe 1 mL (One Med), thermometer, porcelain cup, pot t3, centrifuge (Hettich EBA 200, Kirchlengern, Germany), stative, clamp, mortar and pestle, filler bulbs, Franz diffusion cell, water bath (HH-S2), sonicator (Branson 1800, Brookfield, CT, USA), oven (Memmert, Schwabach, Germany), UV–Vis spectrophotometer (Shimadzu 1280, serial A120660, Kyoto, Japan), FTIR spectrophotometer (Perkin-Elmer UATR Spectrum Two, Waltham, MA, USA), SEM-EDX (JEOL JED-2300, Tokyo, Japan), and pH meter (Thermo Scientific, Waltham, MA, USA).

### 2.3. Synthesis of Molecularly Imprinted Polymer (MIP-Curcumin) and Non-Molecularly Imprinted Polymer (NIP)

MIP synthesis was carried out using the precipitation polymerization method. The first stage was the dissolution of 0.05 mmol, 0.075 mmol, and 0.1 mmol curcumin in 50 mL acetonitrile. Then, 4 mmol MAA was added and the solution was left to stand for 10 min. Next, 10 mmol EGDMA and 1 mmol BPO were added and stirred until completely dissolved. Dissolved oxygen was removed by degassing using sonication under a nitrogen gas flow for 5 min. The mixture was placed in an oil bath at a temperature of 60–70 °C and stirred for 24 h. After the solution became turbid, it was centrifuged at 4000 rpm for 15 min to separate the solid phase. The solid obtained was dried in an oven at 65 °C for 1 h. To remove curcumin, the solid was sonicated in methanol/acetic acid (6:4) every 1 h until no curcumin was detected. Next, the solid was washed using methanol and DM water until the polymer pH ranged from 4.5 to 6.5. This washing aims to remove residual acetic acid. Then, the polymer was dried. The results of the synthesis and release of curcumin were analyzed using FTIR spectroscopy and SEM-EDX.

The NIP was synthesized using the same method as the MIP, but without the addition of curcumin.

### 2.4. Preparation of Phosphate Buffer Solution pH 7.4

The phosphate buffer solution was prepared by mixing 8 mL of 0.02 M NaH_2_PO_4_ solution and 29 mL of 0.01 M Na_2_HPO_4_ solution in 100 mL of DM water. The mixture was shaken until homogeneous, and the pH was measured to pH 7.4 using a pH meter.

### 2.5. Preparation of Curcumin Standard Curve in 1% Ethanol in Phosphate Buffer Solution

Making a standard curve began with making 1000 ppm curcumin in ethanol. A total of 1 mL of 1000 ppm curcumin standard solution was diluted by adding a phosphate buffer solution to a 100 mL volumetric flask to produce a 10 ppm standard solution. Standard solutions were diluted with varying concentrations of 0 ppm, 1 ppm, 2 ppm, 3 ppm, 4 ppm, 5 ppm, 6 ppm, 7 ppm, 8 ppm, 9 ppm, and 10 ppm. For dilution, the 10 ppm standard solution was pipetted in 1 mL, 2 mL, 3 mL, 4 mL, 5 mL, 6 mL, 7 mL, 8 mL, 9 mL, and 10 mL. Then, it was diluted with a mixture of 1% ethanol in phosphate buffer solution in a 10 mL volumetric flask to the mark. Next, absorbance measurements were carried out using a UV–Vis spectrophotometer at a wavelength of 413 nm. The maximum wavelength was determined by scanning at a wavelength of 200 to 800 nm.

### 2.6. Determination of the MIP Imprinting Factor

Adsorption was carried out using the batch method. A total of 0.1 g of the MIP of all variations and the NIP was contacted with 10 mL of curcumin solution with a concentration of 5 ppm for 24 h using a shaker. The mixture was separated by centrifugation at 4000 rpm for 10 min. The absorbance of the supernatant was measured using a UV–Vis spectrophotometer at a wavelength of 426 nm (λ max curcumin in methanol solvent). The amount of curcumin bound to the polymer (Q) can be calculated using the following Equation (1).

(1) $$Q\;(mg/g)=\frac{\;V(Ci-Cf)}{W}$$

where *V*, *Ci*, *Cf*, and *W* represent the volume of the solution (L), initial concentration of curcumin in the solution (mg/L), final concentration of curcumin in the solution (mg/L), and polymer weight (g), respectively. The imprinting factor was determined using Equation (2).

(2) $$\;\;IF=\frac{Q_{MIP}}{Q_{NIP}}$$

where *Q_MIP_* and *Q_NIP_* represent the adsorption capacities of the MIP and NIP, respectively.

### 2.7. Determination of Contact Adsorption Concentration of Curcumin

A total of 0.1 g of the MIP and NIP was contacted with 10 mL of curcumin solution with varying concentrations of 3 ppm, 5 ppm, 7 ppm, 10 ppm, and 15 ppm for 24 h using a shaker. The mixture was separated by centrifugation at 4000 rpm for 10 min. The absorbance of the supernatant was measured using a UV–Vis spectrophotometer at a wavelength of 426 nm (λ max curcumin in methanol solvent).

### 2.8. In Vitro Test

**Figure 1:**
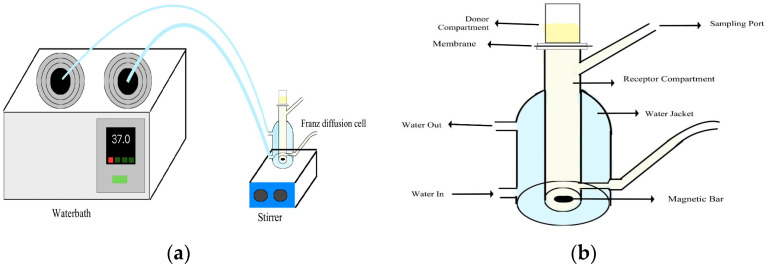
Set-up of (**a**) in vitro testing equipment; (**b**) Franz diffusion cell components.

Initial preparation for the test was carried out by dispersing 0.1 g of the MIP and NIP, which had been adsorbed by a 15 ppm curcumin solution, into 0.4 g of propylene glycol. In vitro testing was carried out using a Franz diffusion cell device and a commercial 0.45 µm semipermeable membrane as an artificial skin membrane. The acceptor compartment of the diffusion cell was filled with phosphate buffer solution pH 7.4. The acceptor compartment temperature was maintained at 37 °C ± 0.5 °C and the stirring speed was 100 rpm. The transdermal formulation was in the donor compartment, where the formulation was placed directly on the membrane. As much as 3 mL of the sample volume in the acceptor compartment was taken at time intervals of 1, 2, 3, 4, 5, 6, 7, and 8 h and replaced with 3 mL of new acceptor fluid. The samples were filtered and analyzed using a UV–Vis spectrophotometer at a wavelength of 413 nm. The test was carried out three times. The set-up of the equipment for in vitro testing and the components of the Franz diffusion cell can be seen in Figure 1.

### 2.9. Characterization of the MIP and NIP

#### 2.9.1. Functional Group Analysis with FTIR Spectrophotometer

The synthesized NIP, MIP-curcumin, and MIP were analyzed using an FTIR spectrophotometer at the Diponegoro University Integrated Laboratory to determine the functional groups contained in the polymers.

#### 2.9.2. Surface Morphology Analysis with SEM-EDX

The synthesized NIP, MIP-curcumin, and MIP were analyzed using SEM-EDX at the Integrated Research and Testing Laboratory at Gajah Mada University to determine the surface morphology and constituent elements of the polymer.

## 3. Results and Discussions

### 3.1. Synthesis of Molecularly Imprinted Polymer

The synthesis of the molecularly imprinted polymer (MIP) was carried out using the precipitation polymerization method. The precipitation polymerization method is a simple polymerization method that involves an excess amount of porogen so that the resulting mixture will be more homogeneous and the resulting polymer has a controlled morphology in the form of uniform particle shapes [10]. In addition, MIP synthesis takes place in situ, where curcumin as a template and methacrylic acid (MAA) as a functional monomer are first contacted with the acetonitrile solvent, then ethylene glycol dimethacrylate (EGDMA) as a cross-linking agent and benzoyl peroxide (BPO) as an initiator are added to initiate polymerization. MAA was used as a functional monomer because it has the ability to donate and accept hydrogen bonds [5]. EGDMA was used as a cross-linking agent because it has vinyl groups which undergo addition during the copolymerization process so that it is able to form cross-links with MAA [13]. Meanwhile, acetonitrile was used as a solvent and porogen because acetonitrile has high efficiency, where its polar aprotic nature allows for a greater number of active sites to be available in the MIP [14].

There are two stages in precipitation polymerization, namely pre-polymerization and co-polymerization. In the pre-polymerization stage, bonds are formed between curcumin and the MAA functional monomer, which is characterized by the formation of HO:H bonds which are relatively weak hydrogen bonds. Next, we proceed to the co-polymerization stage which consists of initiation, propagation, and termination. At this stage, MAA and EGDMA form polymers with the help of BPO as an initiator. BPO has two benzoyl groups which can decompose into radicals when exposed to heat. Therefore, BPO can be used as a powerful source of radicals [15]. The free radical source then attacks the C=C double bonds of MAA and EGDMA. During this stage, dissolved oxygen is removed using degassing and the flow of nitrogen gas. This is because oxygen can produce free radicals which can disrupt the polymerization process. Furthermore, the free radicals produced by BPO spread the reaction chain to become the reaction center. The polymerization reaction was continued for 24 h. In general, an illustration of the formation of the MIP can be seen in Figure S1.

**Figure 2:**
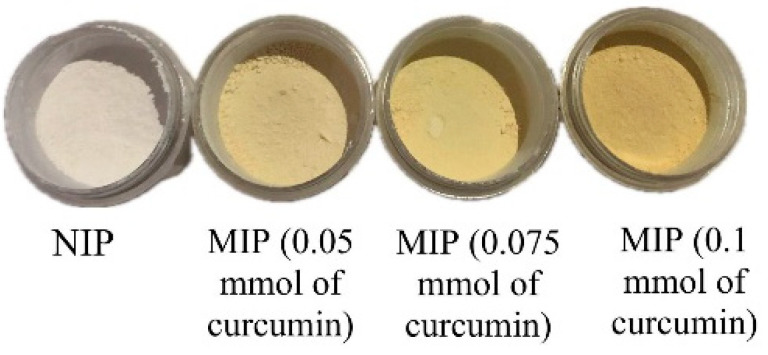
Synthesis results of NIP and MIP-curcumin.

The MIP-curcumin produced was in the form of a yellowish fine powder, and the NIP was a white fine powder, as can be seen in Figure 2.

The difference in polymer color was due to the fact that NIP synthesis does not involve the addition of curcumin. Apart from that, the more curcumin added, the more intense the yellow color of the MIP-curcumin powder will be.

**Figure 3:**
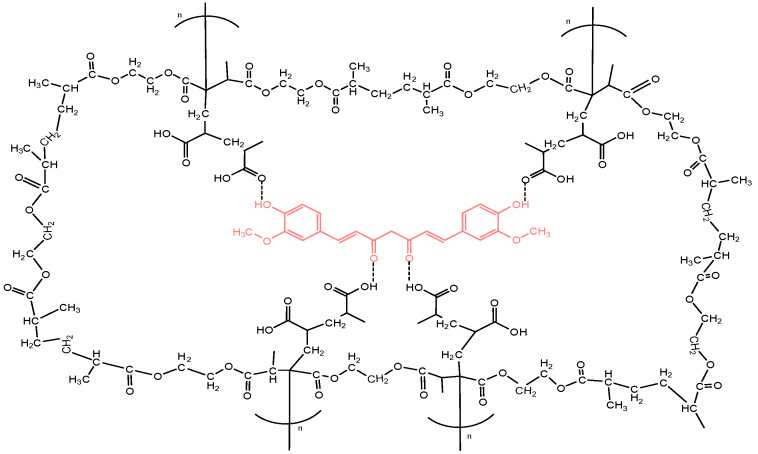
The estimated curcumin–MAA–EGDMA interaction.

The MIP-curcumin and NIP were obtained without crushing so that pore damage can be avoided, as it can disrupt the rebinding process and adsorption ability of the MIP [16]. The estimated interactions between curcumin–MAA–EGDMA can be seen in Figure 3.

The final stage of MIP synthesis was the removal of the curcumin template from the polymer matrix so that a specific binding site or pore would be formed, complementary to the template molecule in terms of size, shape, and ligand. The estimated results of the formed template can be seen in Figure S2.

The process of releasing curcumin from the polymer matrix was carried out using sonication in a mixture of methanol/acetic acid (6:4). Acetic acid is used because it can destroy the hydrogen bonds formed between the functional monomer and the template [17]. Meanwhile, methanol is used because it has high analyte permeability and stronger hydrogen bonds [10]. In this case, methanol is able to weaken the interactions formed between the polymer and the template so that it can elute the curcumin. The filtrate obtained from the removal of curcumin was analyzed using a UV–Vis spectrophotometer at a wavelength of 425 nm (λ max curcumin a mixture of methanol/acetic acid). The absorbance results obtained were converted to ppm using calculations from the linearity equation for curcumin in the solvent methanol/acetic acid (6:4) at a wavelength of 425 nm.

**Figure 4:**
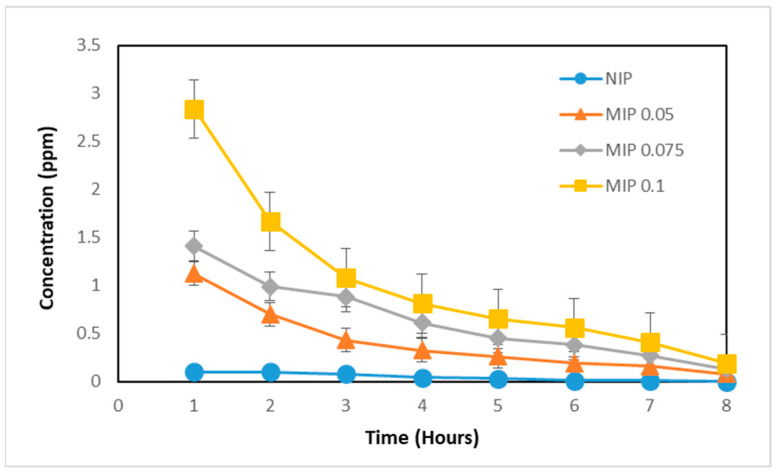
Graph of curcumin removal from polymer matrix.

The release was carried out until no curcumin was detected by the UV–Vis spectrophotometer. The results of curcumin release from the polymer matrix can be seen in Figure 4.

Figure 4 shows that the curcumin concentration decreases over time, indicating that the MIP template has been removed. This is supported by the results of the release from the NIP, which is almost zero, indicating an absence of curcumin in the polymer matrix. Qualitatively, the filtrate from the MIP removal process showed a yellow color, while the NIP wash filtrate was clear. This suggests that curcumin was being removed from the MIP. In addition, Figure 4 shows that the MIP with 0.1 mmol curcumin composition could release more curcumin templates compared to the MIPs with 0.075 mmol and 0.05 mmol curcumin compositions, resulting in more pores complementary to curcumin. The MIP-curcumin removal products were washed using methanol and DM water until the polymer pH ranged from 4.5 to 6.5. This washing aims to remove residual acetic acid which can later affect the rebinding process, and to raise the pH which was originally acidic to a safe pH for medicinal preparations. The neutral MIP was dried and further analyzed using FTIR spectroscopy and SEM-EDX.

### 3.2. MIP and NIP Analysis with FTIR Spectroscopy

**Figure 5:**
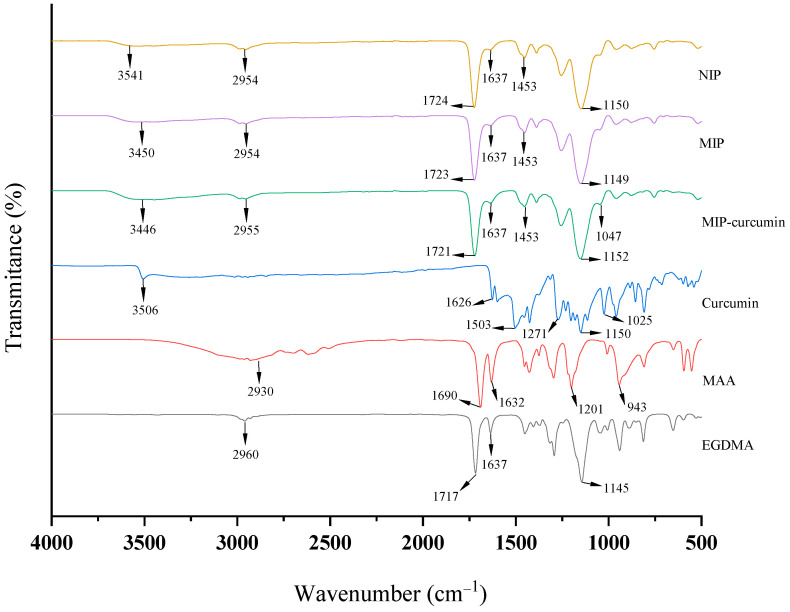
FTIR spectra of the NIP, MIP-curcumin, MIP, and their constituent components.

Characterization using FTIR spectroscopy was carried out with the aim of confirming the success of polymer synthesis by knowing the functional groups contained in the MIP and NIP. The results of FTIR characterization of the NIP and MIP before and after washing, as well as the polymer components consisting of curcumin, methacrylic acid (MAA), and ethylene glycol dimethacrylate (EGDMA), can be seen in Figure 5.

The spectra in Figure 5 show that curcumin has a characteristic peak at 3506 cm^−1^ which indicates -OH stretching vibrations, 1626 cm^−1^ which shows conjugated -C=C vibrations, 1503 cm^−1^ which shows benzene ring vibrations, 1271 cm^−1^ which shows aromatic -CO vibrations, and 1025 cm^−1^ which shows stretching -COC vibrations. For MAA, characteristic peaks can be seen at 2930 cm^−1^ which indicates -CH vibrations, 1690 cm^−1^ which shows -C=O vibrations, 1632 cm^−1^ which shows -C=C vibrations, and 1201 cm^−1^ which shows vibration -CC. EGDMA has a characteristic peak at 1717 cm^−1^ which indicates -C=O vibrations, 1637 cm^−1^ which shows -C=C vibrations, and 1145 cm^−1^ which shows -CO vibrations. The NIP, MIP-curcumin, and MIP have a similar spectrum to EGDMA, where the peak is very clearly visible at 1723 cm^−1^ which indicates -C=O vibrations and 1149 cm^−1^ which indicates -CO vibrations. These two bonds come from the ester group found in EGDMA. This functional group is dominant in the polymer spectrum because EGDMA is the main constituent in the polymer structure [18].

**Figure 6:**
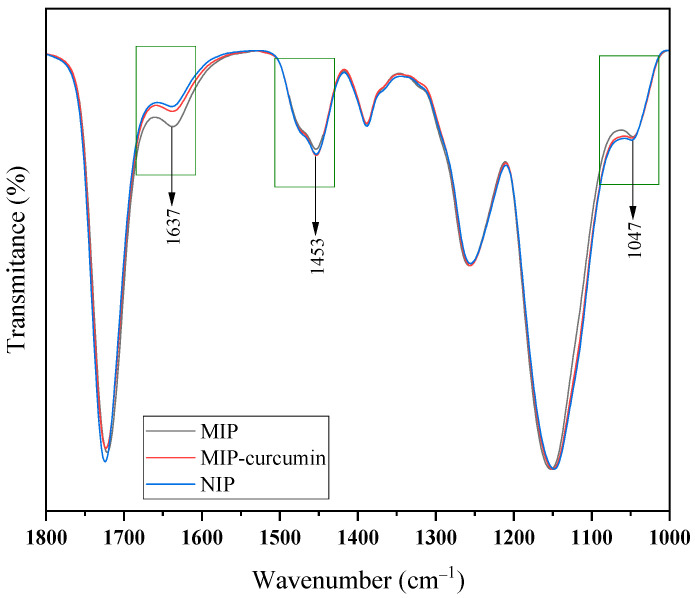
Differences in peak spectra of NIP, MIP-curcumin, and MIP at wave numbers 1000 to 1800 cm^−1^.

The peaks that appear in the NIP, MIP-curcumin, and MIP spectra confirm the presence of MAA and EGDMA in the material. However, the C=C vibration at 1632 cm^−1^ in MAA is reduced in the polymer, especially in the NIP, which confirms the polymerization process, where the double bonds in MAA are broken [18]. The differences in several peaks in the NIP, MIP-curcumin, and MIP spectra can be seen in Figure 6.

Based on Figure 6, the differences in the spectra of the NIP, MIP-curcumin, and MIP are focused on wave numbers 1637 cm^−1^, 1453 cm^−1^, and 1047 cm^−1^. The C=C vibration at 1637 cm^−1^ of the NIP almost disappeared compared to the MIP-curcumin and MIP, which indicates the presence of curcumin in the polymer. However, in the MIP the vibration peak of 1637 cm^−1^ is smaller than in the MIP-curcumin due to washing, which suggests that the curcumin has been released from the polymer. This is in accordance with previous research, where the C=C spectrum in the MIP-curcumin and MIP was clearly observed but in the NIP the peak almost disappeared, indicating the presence of curcumin in the polymer. The -COH vibration at 1453 cm^−1^ in the MIP-curcumin has a smaller intensity compared to the NIP and MIP. This is thought to occur due to the bond formed between MAA and curcumin [19]. In addition, the -COC- vibration at 1047 cm^−1^ in the MIP-curcumin is more prominent compared to the NIP and MIP. This is thought to occur due to the contribution of the ether group contained in curcumin.

### 3.3. MIP and NIP Analysis with SEM-EDX

**Figure 7:**
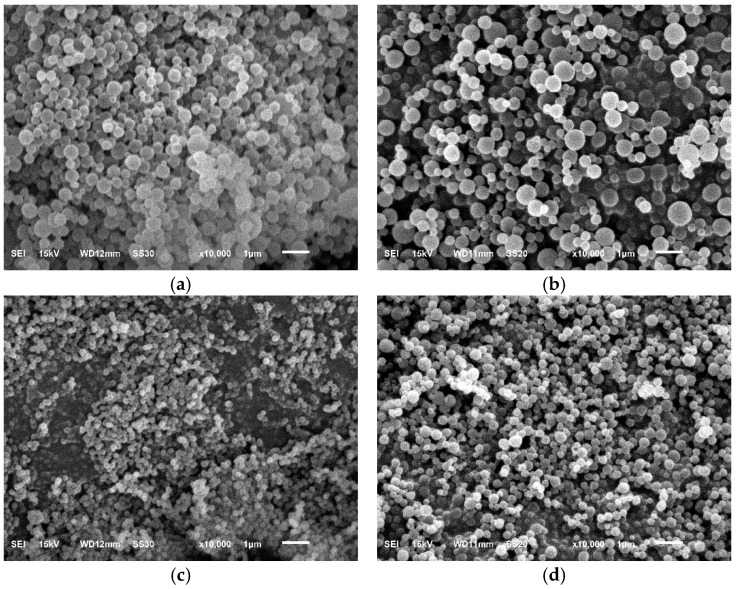
Surface morphology of (**a**) MIP before release, (**b**) MIP, (**c**) NIP before release, (**d**) NIP.

SEM-EDX characterization was carried out to determine the surface morphology of the material as well as the levels of C and O elements contained in the material. The surface morphology of the MIP and NIP before and after release at 10,000× magnification can be seen in Figure 7.

Figure 7 shows that the particles produced by MIP and NIP before and after the removal of curcumin have a uniform spherical shape. This confirms that the precipitation polymerization was successful [10]. Based on the SEM imaging above, it can be seen that NIP has a smaller size than MIP before release. This is thought to be due to the absence of curcumin in the polymer, resulting in smaller granules.

**Figure 8:**
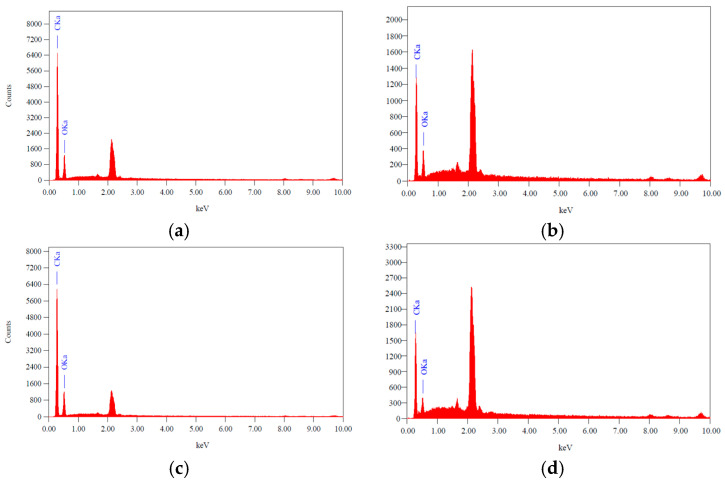
Content of constituent elements of (**a**) MIP before removal, (**b**) MIP, (**c**) NIP before removal, (**d**) NIP.

The levels of polymer constituents in the form of C and O in the material can be determined using EDX analysis. The levels of C and O elements in the material can be seen in Figure 8.

Table 1 shows the results of the detection of elements contained in the NIP and MIP before and after the removal of curcumin. Based on the EDX test results in Table 1, it shows that the C element composition in the NIP before the removal of curcumin is smaller than that of the MIP before the removal of curcumin. This is thought to be due to the contribution of C atoms from curcumin contained in the MIP before the removal of curcumin. Apart from that, in the MIP there is a significant decrease in the mass of C atoms after the removal of curcumin (8.85%). This can confirm that the curcumin has been removed from the polymer matrix. Meanwhile, in the NIP there is also a decrease in the mass of C atoms after the removal of curcumin (3.9%). This is possible because before removal there are still impurities originating from the solvent.

### 3.4. Determination of the MIP Imprinting Factor

**Figure 9:**
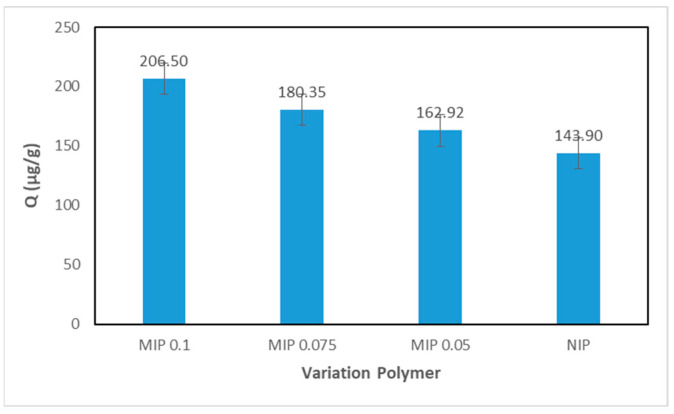
Comparison graph of the adsorption capabilities of the MIP and NIP.

The imprinting factor can be obtained by comparing the adsorption capabilities of the MIP and NIP. In this case, the MIP with a variation in the number of prints of 0.1 mmol, 0.075 mmol, and 0.05 mmol and the NIP were adsorbed in a 5 ppm curcumin solution in methanol using a batch system. A comparison graph of adsorption abilities between the MIP and NIP can be seen in Figure 9.

Based on the graph in Figure 9, the MIP 0.1 mmol has the highest adsorption capacity out of all tested prints. This can happen because during template removal, MIP 0.1 mmol is able to release more curcumin, resulting in many pores becoming available that are complementary to curcumin.

**Figure 10:**
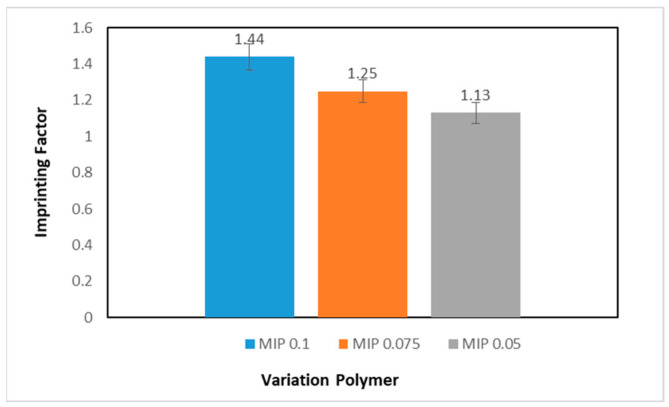
Comparison graph of the number of the MIP prints against the imprinting factor value.

Once the adsorption capacity of the MIP and NIP is known, the imprinting factor (IF) can be calculated. By knowing the IF value, the selectivity of the MIP can be determined. The results of the IF value on the MIP with variations in the number of prints can be seen in Figure 10.

Based on the graph above, it is known that the MIP with an impression amount of 0.1 mmol has the highest IF value. This shows that the MIP with a template composition of 0.1 mmol has better selectivity than the MIP with template quantities of 0.075 and 0.05 mmol. Therefore, the MIP 0.1 mmol will be used further in this research.

### 3.5. Curcumin Adsorption with Varying Curcumin Solution Concentration

**Figure 11:**
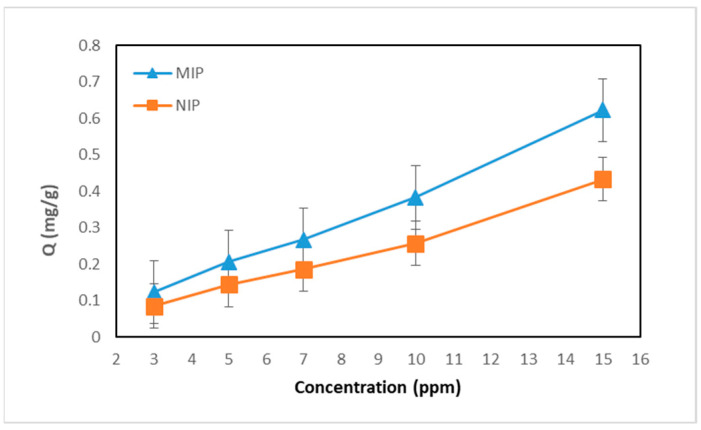
Effect of curcumin solution concentration on the adsorption ability of the MIP and NIP.

Variations in the concentration of curcumin solution during MIP and NIP adsorption were carried out to determine the optimum number of curcumin molecules that could be adsorbed by the MIP and NIP. The concentration of the initial curcumin solution was varied from 3 ppm, 5 ppm, 7 ppm, 10 ppm, to 15 ppm. Contact between the MIP and NIP with the solution was carried out using a shaker for 24 h to allow the curcumin molecules to collide directly with the polymer material so that they could fill the pores formed in the polymer. The supernatant obtained was analyzed using a UV–Vis spectrophotometer at a wavelength of 426 nm. The effect of curcumin solution concentration on the adsorption ability of the MIP and NIP can be seen in Figure 11.

The graph in Figure 11 shows that the amount of curcumin adsorbed by MIP and NIP increases as the initial concentration of the curcumin solution increases. This is because the increasing number of curcumin molecules can interact and collide with the adsorbent. At high concentrations, curcumin is able to occupy many recognition sites on the adsorbent so that it can produce greater adsorption until it reaches saturation. Meanwhile, at low concentrations, curcumin is only able to occupy a small portion of the recognition sites on the adsorbent so that the resulting adsorption is also smaller. Based on Figure 11, it can be seen that the MIP equilibrium state has not been reached at a concentration of 15 ppm. This allows the concentration of the initial solution to be increased until a saturation state is reached. This saturation state is a state where adsorption will remain constant even though the initial solution concentration continues to be increased [17].

Apart from that, in Figure 11 it can be seen that at all variations in curcumin solution concentration, MIP has a greater adsorption capacity than NIP. This may occur because MIP has better recognition ability and specificity for curcumin due to the presence of abundant printed cavities. In MIP adsorption, two bindings occur, namely specific binding where hydrogen bonds are formed between curcumin and the active site, and non-specific binding. Meanwhile, in the NIP only non-specific binding occurs, where physical adsorption dominates so that less adsorption occurs. At a concentration of 3 ppm, the adsorption between MIP and NIP is not too different. This could indicate that curcumin is adsorbed to non-specific sites first and after some of the non-specific sites are filled, curcumin will start to fill specific sites. So, at higher concentrations, non-specific sites will be filled more quickly and binding to specific sites will be more efficient.

### 3.6. Determination of Adsorption Capacity

The higher the concentration of the initial solution, the more curcumin will be adsorbed until equilibrium is reached. Figure 11 shows that the adsorption capacity of the MIP continues to increase to the highest concentration, namely 15 ppm, so the adsorption capacity can be determined using the Langmuir and Freundlich isotherm approach. The linear modeling of the Langmuir isotherm can be obtained through Equation (3).

(3) $$\frac{1}{qe}=\frac{1}{Klqm}\times \frac{1}{Ce}+\frac{1}{qm}$$

where *qe* represents the amount of adsorbed molecules, *K_L_* is the Langmuir constant, *Ce* is the equilibrium concentration of the adsorbate, and *qm* represents the maximum adsorption capacity.

The linear and non-linear modeling of the Freundlich isotherm can be obtained through Equation (4).

(4) $$\log qe=\frac{1}{n}logCe+\log Kf$$

where *qe* represents the amount of adsorbed molecules, *Kf* is the Freundlich constant, *Ce* is the equilibrium concentration of the adsorbate, and *n* is the empirical constant [20].

**Figure 12:**
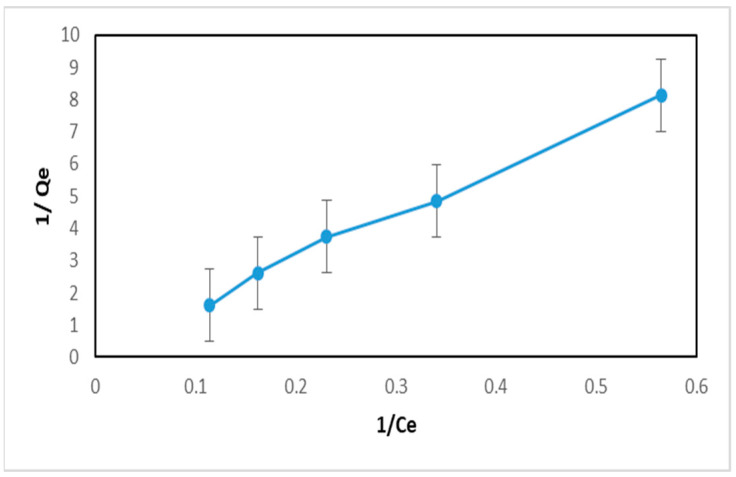
MIP Langmuir adsorption isotherm curve.

**Figure 13:**
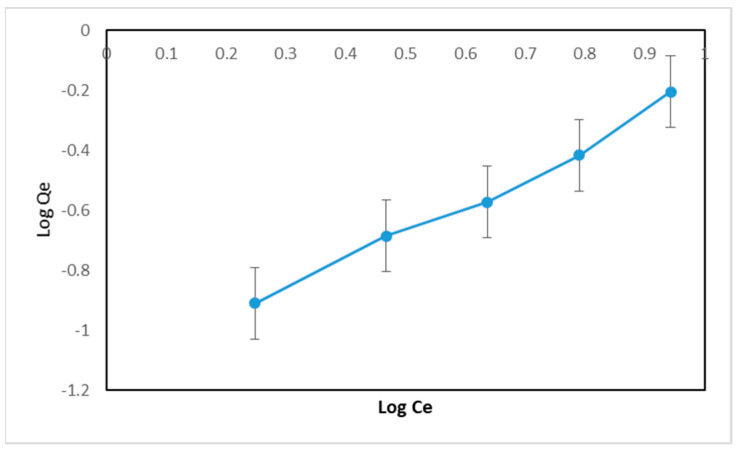
Freundlich MIP adsorption isotherm curve.

The appropriate adsorption isotherm model is determined by the linearity of the curve. In the Langmuir isotherm, the linearity of the curve is obtained from the relationship between 1/Ce and 1/qe. Meanwhile, in the Freundlich isotherm, the linearity of the curve is obtained from the relationship between log*Ce* and log*qe*. The Langmuir and Freundlich isotherm curves from the MIP can be seen in Figure 12 and Figure 13.

Based on Figure 12 and Figure 13, it can be seen that the correlation coefficient for the Langmuir isotherm in the MIP is 0.9935 and for the Freundlich isotherm it is 0.9877. The correlation coefficient of the Langmuir isotherm is closer to 1 than the Freundlich isotherm. Therefore, the Langmuir adsorption isotherm is more suitable for determining the adsorption capacity of the MIP. In general, the adsorption isotherm model and its parameters can be seen in Table 2.

*K_L_* is the Langmuir constant, *qm* is the maximum capacity (mg/g), *K_F_* is the Freundlich constant, *n* is the empirical constant, and *r* is the correlation coefficient.

Table 2 shows that the MIP and NIP both follow the Langmuir isotherm model. This isotherm model shows that the adsorbent contains homogeneous sites. Theoretically, the sites on this adsorbent are selective. However, in practice some non-selective sites remain present in the printed matrix. Based on Table 2, the maximum capacity of the MIP (4.239 mg/g) is greater than the NIP (3.219 mg/g). This is because in the MIP, there are selective recognition sites for printed particles in addition to non-selective sites. Meanwhile, in the NIP, the curcumin molecule will be adsorbed on non-selective sites to a certain extent, but with a different affinity to the selective recognition site of the MIP. This can be seen from the KL value of the MIP (0.016 L/mg) which is greater than that of the NIP (0.012 L/mg), so that the affinity of the MIP will also be greater than that of the NIP. Apart from that, printing efficiency can be determined through the ratio of qm MIP/qm NIP, where in this study a value of 1.317 was obtained.

### 3.7. In Vitro Test

**Figure 14:**
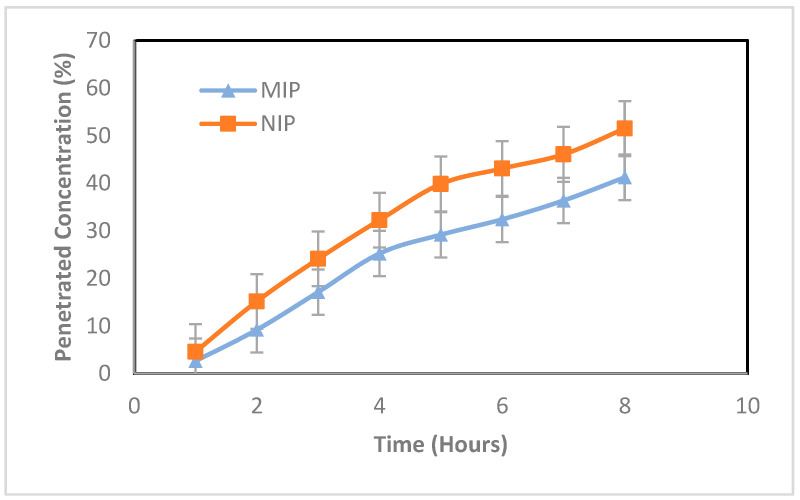
Comparative graph of the MIP and NIP in vitro test results.

The release of curcumin was evaluated by adding propylene glycol to the polymer particles. Propylene glycol acts as a vehicle and enhancer that can increase the penetration of drug preparations [21]. The synthesized polymer is hydrophobic, so that when added with propylene glycol, the polymer does not dissolve and is dispersed. Evaluation of curcumin release in the MIP and NIP was carried out using a Franz diffusion cell for 8 h. Every hour, samples from the receptor compartment were taken and analyzed using a UV–Vis spectrophotometer at a wavelength of 413 nm. The results of the in vitro curcumin release test on MIP and NIP can be seen in Figure 14.

Based on the graph in Figure 14, the percentage of curcumin release at the 8th hour from the MIP (41.26%) is lower than that from the NIP (51.50%). This shows that printing polymers are able to influence the diffusion of active substances so that they can provide a more controlled release compared to unprinted polymers. Printing on MIPs can help extend the half-life of drugs and increase therapeutic efficiency [14].

In addition, the release kinetics of curcumin from the MIP and NIP was carried out to determine the appropriate release kinetic model. In transdermal preparations, there are three release kinetic models that are commonly used, namely zero order, first order, and Higuchi model kinetics [22]. The appropriate kinetic model is determined from the linearity of the curve. At zero order, linearity is determined by the percent drug release over time. In first order, linearity is determined by the ln percent of drug remaining over time. And for the Higuchi model, linearity is determined by the percentage of drug release over the square root of time. The results of the kinetic analysis of curcumin release from the MIP and NIP can be seen in Table 3 and Figure S3.

Where R is the correlation coefficient, K is the rate constant, and SD is the standard deviation (*n* = 3).

Table 3 shows that both the MIP and NIP follow the Higuchi release kinetics model because their correlation coefficients are higher compared to zero-order or first-order models. Drug release following the Higuchi model is dependent on the diffusion of curcumin from the polymer matrix [23]. Additionally, this kinetic model indicates that drug release is proportional to the square root of time. Therefore, the release rate of the drug decreases as time increases.

Referring to the difference in the percentage of curcumin released from the MIP and NIP, it can be seen that the difference occurs due to the influence of imprinting on the MIP. Printing on the MIP can modify the diffusion of drugs from the polymer matrix, resulting in a decrease in the release rate of the MIP [22]. The Higuchi order constant value for the MIP (21.372 ± 0.9) is smaller than that for the NIP (25.836 ± 0.69). Therefore, the MIP is able to provide a more controlled and longer release so that it can be used as a candidate for transdermal delivery of curcumin.

To prove the difference in the percentage of MIP and NIP releases, a *t* test was carried out. Based on the *t* test calculations in the attachment, it is known that the calculated t is 3.832 and the t table at the 95% confidence level is 2.92. The calculated t value is greater than the t table, so that H0 is rejected and the average percentage of MIP release at the 8 h is significantly smaller than that of the NIP.

## 4. Conclusions

The synthesis of curcumin-imprinted polymers (MIPs) was successfully carried out through precipitation polymerization, resulting in polymers with a controlled morphology. FTIR studies indicated the presence of EGDMA and MAA in the polymer matrix and confirmed the occurrence of the polymerization process. The specific recognition capability of the MIP can enhance the adsorption capacity compared to non-imprinted polymers (NIPs). Based on this study, the MIP shows promise for transdermal delivery of curcumin. Imprinting in the MIP can modify drug diffusion from the polymer matrix, leading to a reduction in release rate, which can improve therapeutic efficiency. Therefore, the MIP provides a more controlled and prolonged release, making it a potential candidate for the transdermal delivery of curcumin. The author’s suggestion is that the final preparation can be modified into a patch form so that it is more efficient in use.

## Figures and Tables

**Table 1:** Detection of elements in MIP and NIP before and after the removal of curcumin.

Sample	Mass (%)		Atoms (%)	
	C	O	C	O
MIP before removal	67.83	32.17	73.75	26.25
MIP	58.98	41.02	65.70	34.30
NIP before removal	66.97	33.03	72.98	27.02
NIP	63.07	36.93	69.47	30.53

**Table 2:** Linear adsorption isotherm model and its parameters.

Polymer	Langmuir Isotherm			Freundlich Isotherm		
	*K_L_*	*qm*	*R*	*K_F_*	*n*	*r*
MIP	0.016	4.239	0.9935	0.069	1.025	0.9877
NIP	0.012	3.219	0.9941	0.039	1.027	0.9871

**Table 3:** Results of kinetic analysis of curcumin release from the MIP and NIP.

Polymer	Parameter	Kinetic Model		
		Zero Order	Order One	Higuchi
MIP	R	0.972	0.988	0.993
	K ± SD	5.425 ± 0.2	0.0712 ± 0.004	21.372 ± 0.9
NIP	R	0.959	0.985	0.992
	K ± SD	6.515 ± 0.15	0.095 ± 0.004	25.836 ± 0.69

## Data Availability

The original contributions presented in this study are included in the article. Further inquiries can be directed to the corresponding author.

## Supplementary Materials

The following supporting information can be downloaded at: https://www.mdpi.com/article/10.3390/polym16243456/s1, Figure S1. Illustration of the Process of Imprinting Curcumin Molecules [19]. Figure S2. The estimated results of curcumin imprinting on MIP. Figure S3. Results of Adsorption Kinetics with the model (a) Zero Order of MIP; (b) Zero Order of NIP; (c) First Order of MIP; (d) First Order of NIP; (e) Higuchi of MIP; (f) Higuchi of NIP.
