# Supplementary material for: Precipitation Polymerization-Based Molecularly Imprinted Polymers: A Novel Approach for Transdermal Curcumin Delivery
Source: Polymers (Basel). 2024 Dec 10;16(24):3456. doi: 10.3390/polym16243456 (PMC11678942; doi:10.3390/polym16243456)
Supplement: Supplementary file 1 [file polymers-16-03456-s001.zip › polymers-3299816-supplementary.pdf]

ELECTRONIC SUPPLEMENTARY DATA

Precipitation Polymerization-Based Molecularly Imprinted Polymers: A Novel Approach for Transdermal Curcumin Delivery

Muhammad Cholid Djunaidi [1,*], Viona Resda Putri [1], Nesti Dwi Maharani [1], Retno Ariadi Lusiana [1], Parsaoran Siahaan [1] and Sunarno Sunarno [2]

[1] Department of Chemistry, Faculty of Science and Mathematics, Diponegoro University, Jl. Prof. Soedharto SH, Tembalang, Semarang 50275, Indonesia
[2] Department of Biology, Faculty of Science and Mathematics, Diponegoro University, Jl. Prof. Soedharto SH, Tembalang, Semarang 50275, Indonesia
* Correspondence: cholidjunaidi@live.undip.ac.id

ORCID: http://orcid.org/0000-0003-2594-5062

TABLE OF CONTENTS

S1: Illustration of the Process of Imprinting Curcumin Molecules **(S-3)**

S2: The Estimated Results of Curcumin Imprinting on MIP **(S-5)**

S3: Results of Adsorption Kinetics with the Model **(S-6)**

S4: References **(S-8)**

S1: ILLUSTRATION OF THE PROCESS OF IMPRINTING CURCUMIN MOLECULES

There are two stages in precipitation polymerization, namely pre-polymerization and co-polymerization. In the pre-polymerization stage, bonds are formed between curcumin and the MAA functional monomer, which is characterized by the formation of HO:H bonds which are relatively weak hydrogen bonds. Next, we proceed to the co-polymerization stage which consists of initiation, propagation and termination. At this stage MAA and EGDMA form polymers with the help of BPO as an initiator. BPO has two benzoyl groups which can decompose into radicals when exposed to heat. So BPO can be used as a powerful source of radicals [15]. The free radical source then attacks the C=C double bond of MAA and EGDMA. During this stage, dissolved oxygen is removed using degassing and the flow of nitrogen gas. This is because oxygen can produce free radicals which can disrupt the polymerization process. Furthermore, the free radicals produced by BPO spread the reaction chain to become the reaction center. The polymerization reaction will continue continuously for 24 hours until the reaction is complete or there are no more free radicals. In general, an illustration of the formation of MIP can be seen in Figure S1.

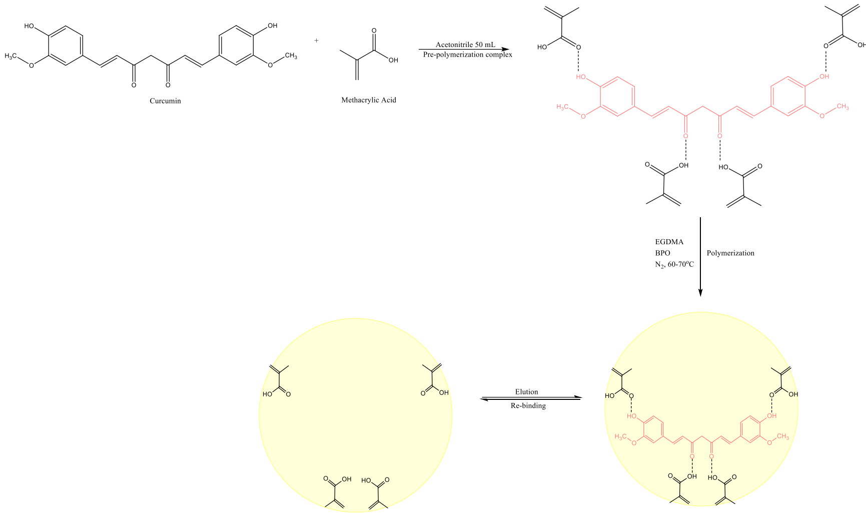

Figure S1. Illustration of the Process of Imprinting Curcumin Molecules [19].

S2: THE ESTIMATED RESULTS OF CURCUMIN IMPRINTING ON MIP

The final stage of MIP synthesis is the removal of the curcumin template from the polymer matrix so that a specific binding site or pore is formed that is complementary to the template molecule in terms of size, shape and ligand. The estimated results of the formed template can be seen in Figure S2.

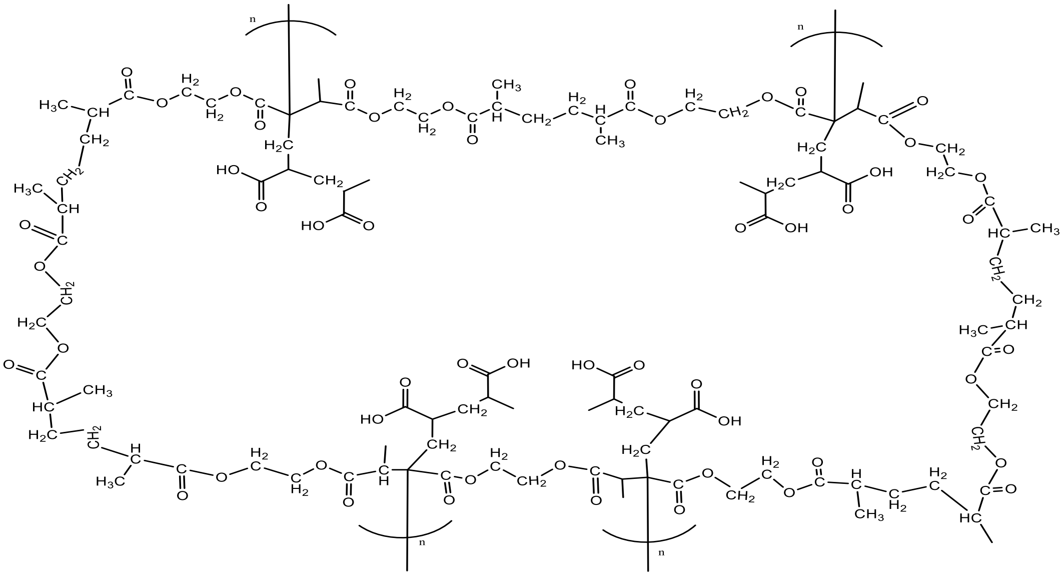

Figure S2. The estimated results of curcumin imprinting on MIP.

S3: RESULTS OF ADSORPTION KINETICS WITH THE MODEL

In addition, the release kinetics of curcumin from MIP and NIP was carried out to determine the appropriate release kinetic model. In transdermal preparations, there are three release kinetic models that are commonly used, namely zero order, first order and Higuchi model kinetics [22]. The appropriate kinetic model is determined from the linearity of the curve. At zero order, linearity is determined by the percent drug release over time. In first order, linearity is determined by ln percent of drug remaining versus time. And for the Higuchi order, linearity is determined by the percentage of drug release at the root of time. The results of kinetic analysis of curcumin release in MIP and NIP can be seen in Table 3 and S3.

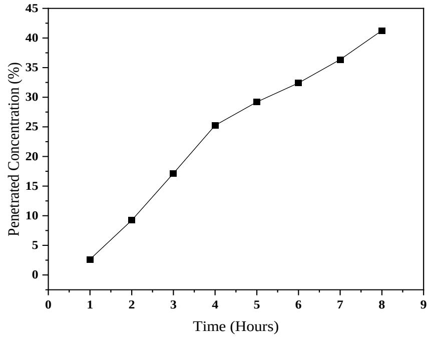

(a)

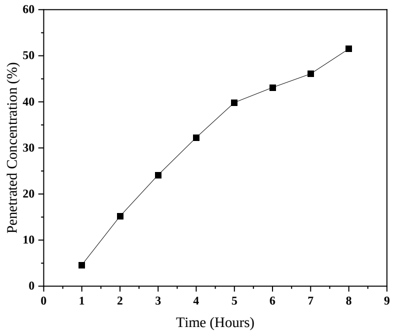

(b)

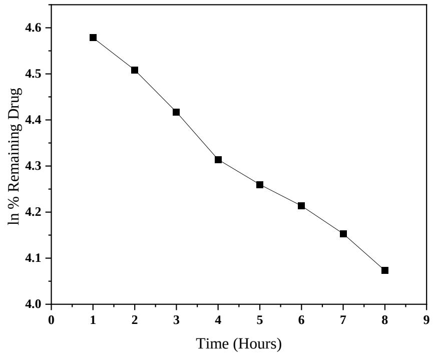

(c)

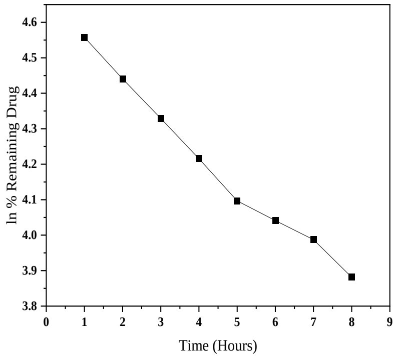

(d)

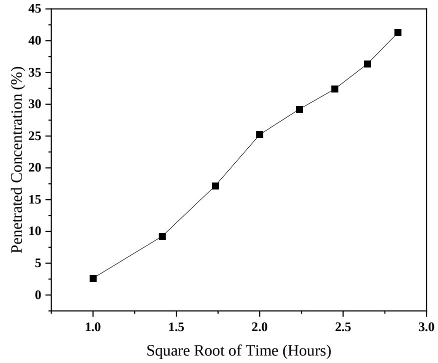

(e)

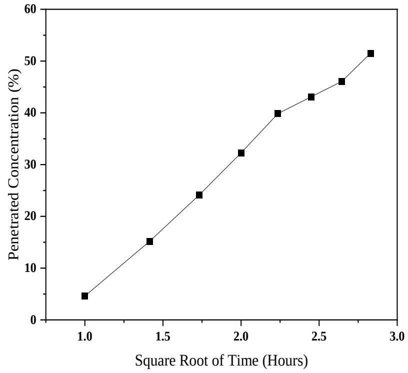

(f)

Figure S3. Results of Adsorption Kinetics with the model a.) Zero Order of MIP; b.) Zero Order of NIP; c.) First Order of MIP; d.) First Order of NIP; e.) Higuchi of MIP; f.) Higuchi of NIP.

S4: REFERENCES

15. Amor, N.; Noman, M.T.; Petru, M. Classification of textile polymer composites: Recent trends and challenges. *Polymers* **2021**, *13*, 2592.

19. Nguyen, V.-T.; Giang, H.H.; Tran, T.N.; Van, T.-K.; Tran, T. Synthesis and Characteristics of Polymer-Mediated Curcumin Molecular Imprinting for Quantitative Determination of Curcumin in Food Samples. *Journal of Chromatography A* **2024**, *1713*, 464567.

22. Lusina, A.; Cegłowski, M. Molecularly imprinted polymers as state-of-the-art drug carriers in hydrogel transdermal drug delivery applications. *Polymers* **2022**, *14*, 640.
